# Supplementary material for: Overmassive black holes and little red dots naturally form in simulations
Source: Nature. 2026 Sep 16;657(8132):621–5. doi: 10.1038/s41586-026-10985-8 (PMC13581593; doi:10.1038/s41586-026-10985-8)
Supplement: Supplementary file 1 [file 41586_2026_10985_MOESM1_ESM.pdf]

nature portfolio

Peer Review File

Overmassive black holes and little red dots naturally form in simulations

Corresponding Author: Dr Sunmyon Chon

Any redactions in this file are there to maintain patient confidentiality, the confidentiality of unpublished data, or to remove third-party material.

This file contains all reviewer reports in order by version, followed by all author rebuttals in order by version.

Version 0:

Reviewer comments:

Referee #1

(Remarks to the Author)
The authors investigate the emergence of LRD-like systems in the high redshift Universe by following the formation of a supermassive star (or quasistar) in a halo irradiated by an intense Lyman-Werner flux from a nearby galaxy. The supermassive star(s) attain ZAMS masses of over 1e5 Msolar masses and subsequently directly collapse into massive black hole seeds. The seeds initially accrete at super-Eddington accretion rates and their host host halo merges with the source (of the LW field) halo. By z ~ 8 the black holes have masses of approximately 1e7 Msolar and reside in haloes with stellar masses of ~ 1e9. The seeds initially grow inside dense envelopes of gas and have characteristics which resembled the recent theories put forward on how LRD are characterised (namely the so-called BH* systems).

The results are indeed novel/unique in the sense that these are the first results to follow the emergence of a BH system from birth of the heavy seed via the SMS stage all the way down to z ~ 8. The BHs formed undergo mergers and accretion of gas showing both super-Eddington and Eddington limited growth pathways. Moreover, the galaxy in which the seed BH forms has characteristics which may indeed resemble an LRD type system.

However, there are also aspects of the work which limit the robustness of the results. Firstly the authors follow an extremely small volume at high resolution and effectively capture only the BH formation and stellar growth in the host halo. No other haloes (including the source of incident LW radiation) are followed self-consistently using the radiation magnetohydrodynamic code. Hence, the statistical relevance of the study is severely compromised and for a Nature paper this is of significant concern. Secondly, it is manifestly unclear that the system the authors describe resembles an LRD system relative to the observational interpretations. Their BH mass is likely at the lower end of inferred LRD masses and moreover the super-Eddington phases that the authors model occur at redshift well beyond (z ~ 13, their Figure 1) current observations of LRD (typically z ~ 4 - 8).

I have a host of other comments on the paper including:

Figure 2: I don't understand why the BH mass a constant mass of 1e3 Msolar as the stellar mass grows. This is not explained or referred to.
Applying a 1e5 K spectrum for the incident LW spectrum is controversial. For example Agarwal et al. 2016 using a 10000 K spectrum indicate that the intensity required is much higher than the 1000 Jcrit values advocated for in this study. In this study the author employ a 100,000 K spectrum (presumably due to PopIII stars) which typically requires even higher intensity levels than the 10000K spectrum. Moreover typing to a black body spectrum is not robust (e.g. Wolcott-Green et al. 2017). In addition to this the spectrum employed by the authors is not self-consistent and is applied isotropically further weakening the model.
Very scant description is given on how accretion is following onto the proto-star in their fiducial simulations
Insufficient descriptions are given for how the modelling of PopIII is performed.
The modelling of the light black hole (LBH) is not well defined.

While most of the above more minor comments can be addressed in the manuscript to some extent the more major concerns

I highlighted above (statistical significance and LRD interpretation) cannot be addressed by updates to the text. Unfortunately I can therefore not recommend that this manuscript be accepted for publication in Nature.

Referee #2

(Remarks to the Author)
A. The manuscript by Chon and collaborators “Rapid emergence of overmassive black holes in the early Universe” addresses the formation and early growth of massive black hole seeds in the Universe and shows very clearly how it is possible to reach the observed masses when seeds form heavy (in haloes exposed to a strong LW flux) and thanks to a brief phase of super-Eddington accretion, which make them appear as LRD-like systems.
B/C/E/H. The manuscript addresses a very timely topic and is well written. The work is original and highly significant. The approach followed is novel, as it combines semi-analytic models with very detailed numerical simulations able to follow the formation of stars and the early growth of MBHs. The results are convincing, and provide important information for our understanding of MBHs at high redshift.
G. The references are appropriate, even though some are missing (see below).
F. Before suggesting it for publication, however, there are some aspects that require clarification, described in the following.

Main concerns:
1) The authors refer to the region they simulated as an overdense proto-cluster region. By looking at the original paper by Ishiyama+21, I could not find any explicit description of the 16 cMpc/h box as being an overdense region of the Universe, which suggests it is an average density region at high redshift. How sure are the authors about the box targeting a protocluster region? Have they measured the cosmological overdensity around the main galaxy in the box?

2) At lines 54-55, the authors claim that after settling at the centre of the main galaxy, the MBH accretes at sub-Eddington up to Eddington rates. This would mean that X-ray emission should be detected, whereas in most observed high-redshift AGN X-rays are not detected, suggesting heavy obscuration of intrinsically X-ray weak spectra (consistent with super-Eddington accretion). For a proper comparison, an analysis of the obscuration might be useful.

3) From the manuscript, it is not clear whether the dense circum-BH disc forms after the MBH has settled in the main galaxy or while it is still hosted by its progenitor halo. In the second case, since no dust is expected to exist in the halo, Compton thick gas would be needed to absorb X-rays from the accretion disc corona, unless the MBH accretes above the Eddington limit (Madau & Haardt 2024). Have the authors checked whether these conditions are satisfied in their simulation (for different viewing angles)?

4) The authors show in the manuscript that through a brief phase of super-Eddington accretion their MBH can grow up to 10^7 Msun in a very short time. However, their model only includes radiative feedback, which is known to be weak due to photon trapping in super-Eddington accretion discs. GRMHD simulations have suggested that in reality vertical advection and strong magnetisation can result in strong outflows that might slow down MBH growth, especially in low-mass haloes (Regan+19, Massonneau+23)

5) In the methods section, the authors describe their semi-analytic approach, in which metal enrichment is only internal to the halo. In the common synchronised pair scenario, the distance between the star-forming halo and the DCBH halo is assumed to be <~1 kpc, to guarantee a high enough LW flux. In this case, instead, the halo is at 10 kpc, which means that the star-forming halo is much more massive and has already formed many stars. Some of these stars should have already enriched the surrounding gas. I am wondering whether the assumption of internal enrichment only might positively bias the abundance of DCBH host haloes in the semi-analytic model used in this work (see Habouzit+16 for simulations about metal enrichment in low-mass haloes, or works by Valiante and collaborators, e.g. Trinca+22, where at least an average external enrichment is considered).

6) In the thermodynamic model employed in the simulations, there are some aspects that require clarifications. First, the authors included X-ray radiation from nearby stars and BHs. I am wondering whether secondary ionisations and secondary heating are also included, as they dominate over the primary interaction (see works by Shull+85 and Dalgarno+99). Secondly, they consider self-shielding following Wolcott-Green+11. Since J21 is applied uniformly on each cell, self-shielding would require an effective length which is much larger than the cell size to properly estimate the radiation attenuation (see the discussion by Safranek-Schrader+17, where the authors explicitly show that attenuation on the cell size highly underestimates absorption). It is not clear, also, how radiation hydrodynamics is implemented in the simulation. Is it on-the-fly RT or an effective model? In the first case, a better approach for the modelling of J21 would have been to inject photons at the edge of the high-resolution region and let the RT solver propagate them consistently. Obviously, this would be expensive to perform, hence I am not asking the authors to do it, but at least they should clarify how radiation attenuation is accounted for in detail. I want to stress that these details might have crucial consequences on their final results, as the formation of H2 and the subsequent fragmentation are highly sensitive to H2 self-shielding. Finally, a few more details about the chemical network might help the reader. For instance, it is not clear whether H2 photodissociation also accounts for the dissociation into H+H+ after H2 photoionisations or not, and whether both direct dissociation and the Solomon process have been included. Moreover, it might be useful to discuss how the network compares with that in Glover 2015, where an extensive discussion about the minimum networks required to properly study the impact of J21 in simulations of PopIII star and DCBH formation.

7) At line 384, the authors claim that, compared to the standard scenario of DCBH formation, in their simulation the collapse

is delayed significantly, resulting in a much more massive halo and the formation of a more massive seed which then grows super-Eddington. The authors should explain in detail the physical reasons responsible for this delay. Turbulence? Dynamical heating? Stronger impact of photo heating that balances atomic cooling? I think this delay explains most of the results in the manuscript, hence the authors should clearly state whether this result is robust or if there are numerical aspects that favoured it.

Minor comments:

a) Line 52: the authors find a MBH-galaxy mass ratio as 1%, and claim that this is one order of magnitude above the local relation. Which relation have they considered as reference? The commonly employed one by Reines & Volonteri 2015 refers to low-luminosity AGN, hence is biased towards low masses, whereas evidence is increasing about the Kormendy & Ho 2013 relation being more representative of the active AGN population.

b) Eq. 4: the piecewise equation reported is not continuous at 2MdotEdd, where the first piece reaches 2LEdd, whereas the second one reaches 2*LEdd(1+log10(2))~ 2.6LEdd, which is 30% higher. It might simply be a typo, where a factor 1/2 is missing in the second piece inside the logarithm. If this is not the case, and the equation has been used in the simulation, the results might be affected by this discontinuity.

c) Robustness tests: The authors claim that a reduction in FUV flux does not significantly change their conclusion. From the manuscript, it seems that the reduction in FUV flux is simply obtained by lowering the star formation efficiency in the semi-analytic model. In principle, this is reasonable. However, I am worried that the non-linear interplay between different processes might simply alter the star formation history, without explicitly changing the FUV flux, if the model were not re-calibrated appropriately. Naively, I expect that if less stars form at time t due to a lower efficiency, less stellar feedback will be released at t+t_fbk, which will result in more more gas remaining available in the galaxy, and a higher SFR at subsequent times. To clarify whether the model actually produces a lower FUV, the authors might show a plot with the FUV flux reaching the target halo as a function of time/redshift in the different runs.

d) Several works have suggested that the MBH masses inferred for MBHs at high redshift might be overestimated by up to 1-2 orders of magnitude (King+24,Lupi+24b,Lambrides+25,Rusakov+25,Abuter+24,GRAVITY+25). How do the authors results fit in this picture?

Referee #3

(Remarks to the Author)
The cosmological simulation presented here is cutting-edge, adding valuable insight to the developing understanding of how the first supermassive black holes formed. This challenge is accentuated by the recent JWST discoveries of a class of overmassive SMBHs at high redshifts (prominently including the LRDs). The authors present a unified picture of a plausible seeding and growth pathway that is consistent with multiple observations. The paper is very well written, in a nicely concise and engaging way.

The work presented here is impressive, but the novelty is somewhat exaggerated. There is a decades-long pre-history of previous simulations of first SMBH formation (e.g., see the review by Inayoshi et al. 2020, ARA&A, 58, 27). To reflect this pre-history, I'd recommend to remove the phrase "for the first time" from the ABSTRACT, which is over-selling things. That being said, this study does push the envelope in presenting a unified approach, whereas previous studies focused on a sub-set of the relevant physics.

Regarding the methodology, I have two comments/questions:
- It is not entirely clear, after reading the METHODS section, how SN feedback is affecting things. Could the authors please clarify this further? As far as I can see, SN feedback is treated on large scales via a SAM approach (how?), but not locally, in the host systems where the BH seeds form (as all Pop III stars are assumed to have initial masses above 260 M_sun). Again, given the possible disruptive impact of (local) SN feedback, please clarify.
- The numerical representation of "black holes" are sink particles, with an effective size that is orders of magnitude larger than
the actual BH event horizon. That is of course unavoidable, given numerical limitations, but some more direct discussion of this limitation would be good. E.g., the authors show zoom-in results that resolve number densities of 10^8 cc^-3. That is still quite low, in terms of densities where the collapsing gas would reach the opacity limit. How do their zoom-in results compare to figures 5 and 6 in Becerra et al. 2018, MNRAS, 480, 5029 (where higher densities are reached)?

I understand that Nature imposes limits on the number of references. Still, I would urge consideration of these studies:
- The key effect explored here is the formation of heavy seed BHs in the presence of strong LW radiation. This specific scenario was first proposed in Bromm & Loeb 2003, ApJ, 596, 34 (earlier papers had worked out the importance of LW radiation in suppressing H_2 cooling, but the connection to heavy seed formation was not made).
- The authors's discussion here of the broad Halpha emission is really nice and convincing. I suggest to cite the recent Rusakov et al. 2026 LRD study in this context (Nature, 649, 574).
- The authors argue that LBH growth is suppressed. That seems quite plausible. But I suggest to briefly compare with two recent related simulation studies (Jeon et al. 2023, MNRAS, 524, 176; and Mehta et al. 2026, Nat Ast, in press). Regarding the former, they argue that LBH growth is suppressed via negative stellar feedback and the "wandering" of light seeds away

from the dense gas reservoir; do the authors see similar wandering of their LBH?

This concludes my report.

Version 1:

Reviewer comments:

Referee #1

(Remarks to the Author)
The authors have to some extent addressed my main concerns. In addressing my concerns the authors have modelling, at high resolution, a later forming accreting massive black hole which is more representative of those observed by JWST.

In summary the authors model the formation of accreting massive black holes formed via progenitor super-massive stars. The massive black holes grow over time before eventually settling into the centre of a neighbouring massive halo.

The results are novel in that they are among the first to probe the evolution of massive black holes in the early Universe at this resolution.

I am happy to support the publication of the study at this time with the caveat that more self-consistent simulations will be required in the near future rather than such focused zoom simulations.

Referee #2

(Remarks to the Author)
I thank the authors for addressing my comments. I think the manuscript is now much clearer and the caveats of the model have been explained in sufficient details. I also thank the authors for providing evidence that their model works in other haloes.

I only have a couple of additional comments to be addressed before suggesting the manuscript for publication.

1) The overdensity of the region is 3.8. A few more details would be helpful. On which scale has the overdensity been estimated? Moreover, is it determined on the mass distribution or using the galaxy number as commonly done in observations?

2) The chemical network employed neglects He. While this is fine for low-temperature gas (below 10^4 K He is fully neutral and does not affect gas thermodynamics at all), in the presence of PopIII stars (with a 10^5 K spectrum) and BHs, He ionisation could significantly alter gas chemistry and thermodynamics, providing additional cooling near the accreting BH and free electrons that might boost H2 formation when gas cools down. A discussion about the choice of neglecting He in their network should be included in my opinion.

Referee #3

(Remarks to the Author)
Since this is my assessment of the revised version, I will here only briefly comment on the authors's response.

- Regarding concerns with the METHODOLOGY, the authors have done a good job in clarifying and further justifying their numerical approach and physical assumptions. Strengths and weaknesses are now quite transparent, amounting overall to an impressive suite of cosmological simulations.

- The current paper is entering an increasingly crowded field. But I think the authors revised characterization of where the novelty lies is fair ("This is the first demonstration that unifies LRDs to a short-lived, enshrouded phase of heavy-seed formation,...). My expectation is that the paper's unified approach will help to advance this vigorously-debated field, both regarding theoretical work and ongoing/future observational campaigns.

Version 2:

Reviewer comments:

Referee #2

(Remarks to the Author)
I thank the authors for answering all my comments, I am happy to suggest the manuscript for publication.

Open Access This Peer Review File is licensed under a Creative Commons Attribution 4.0 International License, which permits use, sharing, adaptation, distribution and reproduction in any medium or format, as long as you give appropriate credit to the original author(s) and the source, provide a link to the Creative Commons license, and indicate if changes were made.
In cases where reviewers are anonymous, credit should be given to 'Anonymous Referee' and the source.
The images or other third party material in this Peer Review File are included in the article's Creative Commons license, unless indicated otherwise in a credit line to the material. If material is not included in the article's Creative Commons license and your intended use is not permitted by statutory regulation or exceeds the permitted use, you will need to obtain permission directly from the copyright holder.
To view a copy of this license, visit https://creativecommons.org/licenses/by/4.0/

Dear Editor,

Thank you for sending us the referee report of our manuscript entitled "Rapid emergence of overmassive black holes in the early Universe". We have addressed the reviewer's comments and all the changes in the manuscript, which are highlighted in red color. Our response to the referee report is attached here.

Sincerely,
Sunmyon Chon, Shingo Hirano, Tomoaki Ishiyama, Seok-Jun Chang, Volker Springel

Referees' comments:
Referee #1 (Remarks to the Author):

Firstly the authors follow an extremely small volume at high resolution and effectively capture only the BH formation and stellar growth in the host halo. No other haloes (including the source of incident LW radiation) are followed self-consistently using the radiation magnetohydrodynamic code. Hence, the statistical relevance of the study is severely compromised and for a Nature paper this is of significant concern.
Secondly, it is manifestly unclear that the system the authors describe resembles an LRD system relative to the observational interpretations. Their BH mass is likely at the lower end of inferred LRD masses and moreover the super-Eddington phases that the authors model occur at redshift well beyond (z ~ 13, their Figure 1) current observations of LRD (typically z ~ 4 - 8).

Answer:: We thank the referee for raising these important concerns. We agree that the original manuscript focused on one high-resolution target halo and did not sufficiently demonstrate either how representative this system is or whether similar heavy-seed formation can occur at redshifts more directly relevant to the observed LRD population. To address this, we have performed additional calculations based on the larger sample of DCBH candidates identified in Ishiyama & Hirano (2025), and added a new section “DCBH Formation in lower-z universe” together with a figure (Extended data Figure 9) showing the BH population and the mass and accretion histories of the newly followed systems.

In the parent 16 cMpc/h box, we identify 62 candidate haloes selected by the same criteria used for the original target, namely exposure to strong LW radiation (J21 > 1000) and sufficient separation from nearby massive haloes to avoid tidal disruption. The original target should therefore be regarded as one member of a broader candidate population rather than an isolated special case.

To further test whether the same growth channel operates at lower redshift, we selected another candidate that collapses later, at z ~ 15. In this case, we find that 8 BHs form around the target halo and several of them grow rapidly to ~10^7 Msun after experiencing super-Eddington accretion. BH formation continues down to z ~ 8, showing that this channel is not confined to the very earliest epoch but extends into the redshift range of the observed LRD population.

Figure 2: I don't understand why the BH mass a constant mass of 1e3 Msolar as the stellar mass grows. This is not explained or referred to.

Answer:: The main reason why the LBH does not grow is that its host halo has a much shallower gravitational potential. The grey line in Extended Data Fig. 5c shows the halo escape velocity, which remains below 10 km/s. Once the gas in the halo is photo-heated by ionizing radiation, it is therefore easily evaporated, since the sound speed of ionized gas is typically ~10 km/s. This is in clear contrast to the MBH1 and MBH2 cases, where the escape velocities remain well above 10 km/s, allowing the haloes to retain the ionized gas.

To further demonstrate the importance of this photo-evaporation, we have estimated the time evolution of the density, temperature, and the Bondi accretion rate, which is attached at the end of this reply. Due to the photoevaporation, the density around the LBH is kept low ~1 cm^-3, and the Bondi accretion rate onto LBH becomes 10^-8 – 10^-7 Msun/yr, which is much smaller than the Eddington rate. As a result, although the stellar mass in the host continues to grow, the LBH cannot re-accrete a dense gas reservoir and its mass remains around 10^3 Msun. This is consistent with previous studies showing that light seeds formed from Pop III remnants grow inefficiently in shallow haloes because radiative feedback evacuates the gas reservoir (e.g. Alvarez et al. 2009; Jeon et al. 2012).

We have added a new section "Inefficient growth of PopIII remnant BH" to clearly state why the LBH hardly grows in our simulation.

Applying a 1e5 K spectrum for the incident LW spectrum is controversial. For example Agarwal et al. 2016 using a 10000 K spectrum indicate that the intensity required is much higher than the 1000 Jcrit values advocated for in this study. In this study the author employ a 100,000 K spectrum (presumably due to PopIII stars) which typically requires even higher intensity levels than the 10000K spectrum. Moreover typing to a black body spectrum is not robust (e.g. Wolcott-Green et al. 2017). In addition to this the spectrum employed by the authors is not self-consistent and is applied isotropically further weakening the model.

Answer:: We chose a 10^5 K spectrum because the spectrum of an actively star-forming galaxy is expected to be closer to 10^5 K than to a 10^4 K blackbody spectrum (Sugimura et al. 2014; Latif et al. 2014). In particular, Sugimura et al. (2014) showed that the critical intensity is typically J21 ~ 1000 for realistic young stellar populations, which is similar to the value obtained for a 10^5 K spectrum. By contrast, a 10^4 K spectrum is more representative of older stellar populations lacking young blue stars.

As the referee points out, the critical LW intensity is lower for a 10^4 K spectrum than for a 10^5 K spectrum (e.g. Shang et al. 2010), because the stronger infrared component more efficiently dissociates H-, the catalyst for H2 formation. In this sense, adopting a 10^5 K spectrum is a conservative choice for identifying DCBH candidates, because it underestimates the H- dissociation rate and therefore does not artificially make direct collapse easier.

To test the sensitivity of our results to the assumption of an isotropic LW radiation field, we performed an additional calculation in which the LW intensity from the source galaxy was

reduced by a factor of 10. Regan et al. (2014) showed that an approximately ten-times higher LW intensity may be required for DCBH formation when the anisotropic distribution of dense gas around the source galaxy is taken into account. We find that the BH system still forms robustly in this case (Extended Data Fig. 10). This suggests that an order-of-magnitude attenuation of the LW intensity, which could plausibly arise from absorption or shielding by the medium between the source galaxy and the DCBH halo, does not prevent DCBH formation in our simulation. Therefore, the heavy-seed formation outcome in our model is robust against at least order-of-magnitude uncertainties in the incident LW field, although we agree that approximating the source spectrum as a blackbody and applying it isotropically is a simplification of the present model.

Very scant description is given on how accretion is following onto the proto-star in their fiducial simulations

Answer:: We thank the referee for raising this point. In the simulation, protostellar accretion is treated using a sink-particle method. Gas cells entering the sink radius are assimilated and their mass is added to the protostar particle. We have now clarified this prescription more explicitly in the first paragraph of the section “Modeling PopIII stars and BHs”, starting with “The mass accretion rate is measured by…” (lines 322-325).

In particular, we state that the mass accretion rate is measured from the gas assimilated by the sink, and that the sink radius is set to 100 times the local cell size, corresponding to approximately 1–20 pc depending on the local resolution.

Insufficient descriptions are given for how the modelling of PopIII is performed.

Answer:: We thank the referee for this comment. The Pop III stellar modelling is already described in the Extended Data, but we agree that this may not have been sufficiently clear from the submitted version. In our model, the stellar properties are assigned by combining pre-calculated stellar evolution tracks for the main-sequence phase and the supergiant protostar phase, depending on the stellar mass and accretion history. Specifically, as stated in the Extended Data (lines 308-318):

“The luminosity and effective temperature of each protostar are determined from its instantaneous mass and accretion rate by interpolating results from one-dimensional stellar evolution models assuming constant accretion rates \citep{Hosokawa+2009}. If the stellar accretion rate exceeds the critical accretion rate of $\dot{M}\text{crit} \equiv 0.02 ~M\odot~\mathrm{yr}^{-1}$, we assume the stellar radius expands due to the injection of the large amount of entropy into the stellar envelope \citep{Hosokawa+2013, Haemmerle+2018, Nandal+2023, Nandal+2025}. During this phase, we assume the surface temperature of the star to be $6000~$K and the luminosity to be the Eddington value following the results of detailed stellar evolution calculations \citep{Hosokawa+2012}. After the accretion rate becomes smaller than $\dot{M}_\text{crit}$, we gradually shrink the stellar radius to the radius expected for main-sequence stars on the timescale of the surface Kelvin-Helmholtz (KH) time, which is given by ten times the stellar KH time \citep{Sakurai+2015}.”

We have changed the subtitle of that section to “Modeling PopIII stars and BHs” to make this description easier to find.

The modelling of the light black hole (LBH) is not well defined.

Answer:: We thank the referee for raising this point. We do not adopt a separate prescription for the LBH and MBHs; they are treated in the same way after BH formation. We have clarified this in the section “Modeling PopIII stars and BHs” at lines 318-322. Once the stellar age exceeds the stellar lifetime, stars with masses above 260 Msun are assumed to collapse directly into BHs following Heger & Woosley (2002). The corresponding star particle is then converted into a BH particle with the same mass.

The BH accretion radius is set equal to the progenitor sink radius if that radius is larger than the Bondi radius; otherwise, it is set to the Bondi radius. The luminosity of the accreting BH is determined by equation (4). For the BH radiation spectrum, we assume a double power-law continuum, with F_nu proportional to nu^-0.6 from 1 to 10 eV and F_nu proportional to nu^-1.5 above 10 eV up to the X-ray band (lines 347 - 366).

~~~~~~

~~~~~~

~~~~~~

~~~~~~

Referee #2 (Remarks to the Author):
Main concerns:
1) The authors refer to the region they simulated as an overdense proto-cluster region. By looking at the original paper by Ishiyama+21, I could not find any explicit description of the 16 cMpc/h box as being an overdense region of the Universe, which suggests it is an average density region at high redshift. How sure are the authors about the box targeting a protocluster region? Have they measured the cosmological overdensity around the main galaxy in the box?

Answer:: We thank the referee for raising this point. We agree that our original wording was not sufficiently precise. By “overdense proto-cluster region,” we did not mean that the entire 16 cMpc/h box is itself an overdense region. Rather, we mean that the zoom-in target lies in an overdense region within the parent box. We have now quantified this more explicitly and find that the overdensity of the zoom-in region corresponds to 3.8 sigma. We have clarified this point in the last sentence in the section “Method” and now avoid wording that could be interpreted as referring to the full 16 cMpc/h box at lines 236 - 237 .

----

2) At lines 54-55, the authors claim that after settling at the centre of the main galaxy, the MBH accretes at sub-Eddington up to Eddington rates. This would mean that X-ray emission

should be detected, whereas in most observed high-redshift AGN X-rays are not detected, suggesting heavy obscuration of intrinsically X-ray weak spectra (consistent with super-Eddington accretion). For a proper comparison, an analysis of the obscuration might be useful.

Answer:: We thank the referee for this important comment. We agree that, once the MBH has settled at the centre of the main galaxy and accretes at sub-Eddington to nearly Eddington rates, the obscuration should be quantified for comparison with observations. In our simulation, we directly measured the hydrogen column density around the BH during this phase and confirmed that the source remains Compton-thick.

As shown in Fig. 4f, gas with nH ~ 10^7-10^8 cm^-3 extends over ~10^4 au around the BH, corresponding to NHI ~ 10^24-10^25 cm^-2. This is true not only on average, but also along the individual sightlines examined in our analysis. Extended Data Fig. 8 shows that the hydrogen column density remains high, NH ~ 10^24-10^25 cm^-2 for all 64 uniformly spaced viewing angles. Therefore, even in the post-settling, sub-Eddington to nearly Eddington phase, the X-ray emission can still be strongly attenuated by the surrounding gas.

This is consistent with recent X-ray constraints on LRDs, where secure individual detections are rare and stacking analyses suggest weaker X-ray emission than expected for unobscured type-I AGN. We have added the last paragraph in the section “Observability of growing MBHs” (lines 539 - 556).

----
3) From the manuscript, it is not clear whether the dense circum-BH disc forms after the MBH has settled in the main galaxy or while it is still hosted by its progenitor halo. In the second case, since no dust is expected to exist in the halo, Compton thick gas would be needed to absorb X-rays from the accretion disc corona, unless the MBH accretes above the Eddington limit (Madau & Haardt 2024). Have the authors checked whether these conditions are satisfied in their simulation (for different viewing angles)?

Answer:: We thank the referee for raising this point. The dense circum-BH disc appears only shortly after BH formation, when the BH is still hosted by its progenitor halo rather than by the main galaxy. We agree that, at this stage, the obscuration should be dominated by gas rather than dust. To check this, we directly measured the hydrogen column density around the BH in the simulation. Extended data Fig. 8 shows that during the early super-Eddington phase at t=26 kyr, the column density is extremely high, above 10^26 cm^-2 for all directions. This is consistent with the dense gas distribution shown in Fig. 4, where the dense gas with n_H~10^11 cm^-3 extends toward the polar direction over a scale of 10^4 au.

These results indicate that, although the gas is dust-free at this stage, the BH is nevertheless heavily obscured by dense neutral gas over a wide range of viewing angles, which is consistent with the recent Chandra stacking results (Sacchi et al. 2025).

To summarize the major points 2) and 3), we have added the last paragraph in the section “Observability of growing MBHs” (lines 539 - 556).

----
4) The authors show in the manuscript that through a brief phase of super-Eddington accretion their MBH can grow up to 10^7 Msun in a very short time. However, their model only includes radiative feedback, which is known to be weak due to photon trapping in super-Eddington accretion discs. GRMHD simulations have suggested that in reality vertical advection and strong magnetisation can result in strong outflows that might slow down MBH growth, especially in low-mass haloes (Regan+19, Massonneau+23)

Answer:: We thank the referee for this important comment. We agree that our current model does not include kinetic feedback such as magnetically driven jets or disk winds, and this is an important limitation of the present study. As the referee points out, GRMHD simulations have suggested that super-Eddington accretion flows can drive strong outflows through vertical advection and magnetic fields, which may reduce the net accretion rate onto the BH, particularly in low-mass haloes.

At the same time, the strength of such kinetic feedback in the early BH growth phase remains uncertain, because it depends on the magnetic field amplification and geometry near the BH, which are not resolved in our simulations. Our current calculation therefore captures radiative feedback, but not the additional suppression of BH growth that may arise from magnetically driven outflows. For this reason, the rapid BH growth found in our model should be regarded as a result obtained in the absence of explicit kinetic feedback, and the final BH mass may be overestimated if strong jets or disk winds are present.

We have added the paragraph in the section “Modeling PopIII stars and BHs” to clarify this caveat and to discuss that kinetic feedback from super-Eddington accretion could further suppress BH growth at lines 367 - 378.

----
5) In the methods section, the authors describe their semi-analytic approach, in which metal enrichment is only internal to the halo. In the common synchronised pair scenario, the distance between the star-forming halo and the DCBH halo is assumed to be <~1 kpc, to guarantee a high enough LW flux. In this case, instead, the halo is at 10 kpc, which means that the star-forming halo is much more massive and has already formed many stars. Some of these stars should have already enriched the surrounding gas. I am wondering whether the assumption of internal enrichment only might positively bias the abundance of DCBH host haloes in the semi-analytic model used in this work (see Habouzit+16 for simulations about metal enrichment in low-mass haloes, or works by Valiante and collaborators, e.g. Trinca+22, where at least an average external enrichment is considered).

Answer:: We expect that the external enrichment should be unimportant because the target halo is located far enough from the star forming galaxy whose separation is around 10 physical kpc (line 426). Dijkstra et al. (2014) have estimated the time evolution of the galactic wind and found that it only extends 1-2 physical kpc within around 300 Myr. Ventura et al. (2024) also statistically studied the distribution of the superbubble around the star forming halo combining N-body simulation and semi-analytic model within the 10 Mpc/h cosmological region, and found that the size of the super bubble is at most 5 physical kpc. These results tell us that the target halo can avoid external metal enrichment.

Furthermore, even if the target halo is metal-enriched, we can still form heavy seed BHs. Chon & Omukai (2020) and (2025) have demonstrated that the formation of the heavy seed BHs in the atomic-cooling halo taken from the cosmological simulation for the different metallicities. They have found that the heavy seed BHs still form the metallicity up to Z/Zsun ~ 10^-3. The metallicity does promote the fragmentation but most of the gas and stars converge to the central massive stars, allowing to form heavy seed BHs in the metal-enriched halo.

For these reasons, we expect that neglecting external enrichment does not qualitatively change the outcome for our target halo, and clarify these points in the 2nd paragraph of the new section “Host halo evolution of MBHs” at lines 433 - 411.

----

6) In the thermodynamic model employed in the simulations, there are some aspects that require clarifications. First, the authors included X-ray radiation from nearby stars and BHs. I am wondering whether secondary ionisations and secondary heating are also included, as they dominate over the primary interaction (see works by Shull+85 and Dalgarno+99). Secondly, they consider self-shielding following Wolcott-Green+11. Since J21 is applied uniformly on each cell, self-shielding would require an effective length which is much larger than the cell size to properly estimate the radiation attenuation (see the discussion by Safranek-Schrader+17, where the authors explicitly show that attenuation on the cell size highly underestimates absorption). It is not clear, also, how radiation hydrodynamics is implemented in the simulation. Is it on-the-fly RT or an effective model? In the first case, a better approach for the modelling of J21 would have been to inject photons at the edge of the high-resolution region and let the RT solver propagate them consistently. Obviously, this would be expensive to perform, hence I am not asking the authors to do it, but at least they should clarify how radiation attenuation is accounted for in detail. I want to stress that these details might have crucial consequences on their final results, as the formation of H2 and the subsequent fragmentation are highly sensitive to H2 self-shielding. Finally, a few more details about the chemical network might help the reader. For instance, it is not clear whether H2 photodissociation also accounts for the dissociation into H+H+ after H2 photoionisations or not, and whether both direct dissociation and the Solomon process have been included. Moreover, it might be useful to discuss how the network compares with that in Glover 2015, where an extensive discussion about the minimum networks required to properly study the impact of J21 in simulations of PopIII star and DCBH formation.

Answer:: We thank the referee for pointing out the importance of the chemical network and radiative transfer treatment. We agree that these details can affect H2 formation and fragmentation, and we have clarified our treatment more explicitly in the revised manuscript. We have expanded the description in section Extended Data “Radiation hydrodynamic calculation” at lines 258 - 264. For the external radiation from the source galaxy, we use a uniform LW background combined with H2 self-shielding. The shielding is not estimated from the local cell size. Instead, we assume that the cloud extends over the local Jeans length and compute the H2 column density as N_H2 = f_H2 n_H l_Jeans, following equation (12) of Wolcott-Green & Haiman (2011). As discussed in that work, using the local Jeans length provides a reasonable approximation, although it tends to overestimate the shielding strength, corresponding to an underestimate of f_sh by about a factor of 2 compared to more

accurate treatments. We chose this method because, although it is less accurate than more elaborate approaches, it is more robust against local density fluctuations than Sobolev-like methods and much less computationally expensive than multi-ray treatments such as the 6-ray method. We have clarified this in the 3rd paragraph of the section “Radiation hydrodynamic calculation” at lines 294 - 296.

Radiation from accreting BHs is treated separately using a ray-tracing method based on RSPH (Susa 2006; Chon et al. 2017). In this calculation, we compute the H2 column density between each BH and each gas cell, and evaluate the shielding factor using the fitting formula of Draine & Bertoldi (1996). For the BH radiation, we include LW, UV, and X-ray photons. We do not include secondary ionization by X-rays in the current model. We agree that this may be important, because secondary ionization enhances the electron fraction, increases the H^- abundance, and thereby promotes H2 formation, which would raise the critical LW intensity for direct collapse. We clarified these separated treatments at lines 268 - 273 and lines 278 - 286.

In our simulation, the X-ray effect is important mainly for the formation of MBH2, but not for MBH1, because no accreting BH is present when MBH1 forms. At the time of MBH2 formation, X-ray irradiation enhances H2 formation, which is seen as a temperature decrease in the green curve of Extended Data Fig. 5b. However, the gas in the outer halo still remains sufficiently warm, so the inflow rate onto MBH2 stays high (Chon & Omukai 2020, 2025). This shows that, in our case, X-ray ionization does not prevent heavy-seed formation.

----

7) At line 384, the authors claim that, compared to the standard scenario of DCBH formation, in their simulation the collapse is delayed significantly, resulting in a much more massive halo and the formation of a more massive seed which then grows super-Eddington. The authors should explain in detail the physical reasons responsible for this delay. Turbulence? Dynamical heating? Stronger impact of photo heating that balances atomic cooling? I think this delay explains most of the results in the manuscript, hence the authors should clearly state whether this result is robust or if there are numerical aspects that favoured it.

Answer:: We thank the referee for raising this point. In our case, the delayed collapse is caused primarily by the slow contraction of gas in a rapidly growing atomic-cooling halo, with an additional contribution from dynamical heating associated with a minor merger. We have added Extended Data Fig. 4, which shows the time evolution of the peak gas density (blue) and the halo mass growth rate (black lines). At t ~ 200 Myr, the halo virial temperature reaches 8000 K and atomic cooling triggers the onset of collapse. However, the density at this stage is still only n_H ~ 3 cm^-3, corresponding to a free-fall time of ~50 Myr, so the gas contraction remains relatively slow and it takes about ~100 Myr for the protostar to form.

In addition, we find a minor merger at t ~ 240 Myr, which further delays the collapse. During the first ~50 Myr after the onset of collapse, the density increases by nearly an order of magnitude, which by itself would shorten the free-fall time. However, the subsequent evolution takes another ~50 Myr to gain a comparable increase in density. We attribute this additional delay to dynamical heating caused by the merger. Although this only delays the collapse by a factor of order unity, the halo mass grows to ~10^8 Msun during this period,

which is what ultimately enables the formation of a more massive seed and the subsequent super-Eddington growth.

We have clarified this interpretation in the last paragraph of the section “Host halo evolution of MBHs” at lines 442 - 463.

Minor comments:

a) Line 52: the authors find a MBH-galaxy mass ratio as 1%, and claim that this is one order of magnitude above the local relation. Which relation have they considered as reference? The commonly employed one by Reines & Volonteri 2015 refers to low-luminosity AGN, hence is biased towards low masses, whereas evidence is increasing about the Kormendy & Ho 2013 relation being more representative of the active AGN population.

Answer:: We compared our result primarily with the relation of Reines & Volonteri (2015). As the referee points out, this relation is based on low-luminosity AGN. We therefore also compared our result with the relation of Kormendy & Ho (2013) (dotted line in Fig. 2), and found that the BH-to-host-stellar-mass ratio in our system is still about an order of magnitude higher than that relation as well.

----

b) Eq. 4: the piecewise equation reported is not continuous at 2MdotEdd, where the first piece reaches 2LEdd, whereas the second one reaches 2*LEdd(1+log10(2))~ 2.6LEdd, which is 30% higher. It might simply be a typo, where a factor 1/2 is missing in the second piece inside the logarithm. If this is not the case, and the equation has been used in the simulation, the results might be affected by this discontinuity.

Answer:: We thank the referee for pointing this out. It was indeed a typo: a factor of 1/2 was missing inside the logarithm in the second branch. We have corrected equation (4) in the revised manuscript.

----

c) Robustness tests: The authors claim that a reduction in FUV flux does not significantly change their conclusion. From the manuscript, it seems that the reduction in FUV flux is simply obtained by lowering the star formation efficiency in the semi-analytic model. In principle, this is reasonable. However, I am worried that the non-linear interplay between different processes might simply alter the star formation history, without explicitly changing the FUV flux, if the model were not re-calibrated appropriately. Naively, I expect that if less stars form at time t due to a lower efficiency, less stellar feedback will be released at t+t_fbk, which will result in more more gas remaining available in the galaxy, and a higher SFR at subsequent times. To clarify whether the model actually produces a lower FUV, the authors might show a plot with the FUV flux reaching the target halo as a function of time/redshift in the different runs.

Answer:: We thank the referee for this important comment. In this robustness test, we did not re-run the semi-analytic model with a different star formation efficiency. Instead, we reduced the incident FUV flux at the target halo while keeping the stellar mass and star formation history from the original semi-analytic calculation fixed. We adopted this procedure

intentionally as a controlled sensitivity test of the collapse outcome to the FUV intensity itself. We agree with the referee that re-running the semi-analytic model with a different efficiency would introduce non-linear changes in the star formation history and feedback, and would therefore probe a different question.

We have clarified this point in the first paragraph of the section “Robustness tests” (lines 599 - 601), so that the scope of this robustness test is stated more explicitly.

----
d) Several works have suggested that the MBH masses inferred for MBHs at high redshift might be overestimated by up to 1-2 orders of magnitude (King+24,Lupi+24b,Lambrides+25,Rusakov+25,Abuter+24,GRAVITY+25). How do the authors results fit in this picture?

Answer:: We agree that virial BH mass estimates at high redshift may be systematically overestimated, possibly by up to 1 dex or more in some cases. In the context of our paper, however, this does not qualitatively change our main conclusion. Even if the observationally inferred BH masses of LRDs were revised downward by about an order of magnitude, our simulated system would still lie in the overmassive BH-to-host regime relevant to the LRD population. In addition, as discussed in the revised manuscript, our estimate of the line broadening and Thomson-scattering optical depth suggests that obscuration and scattering effects may indeed bias simple virial mass estimates upward.
~~~~~~
~~~~~~
~~~~~~
~~~~~~
Referee #3 (Remarks to the Author):

The work presented here is impressive, but the novelty is somewhat exaggerated. There is a decades-long pre-history of previous simulations of first SMBH formation (e.g., see the review by Inayoshi et al. 2020, ARA&A, 58, 27). To reflect this pre-history, I'd recommend to remove the phrase "for the first time" from the ABSTRACT, which is over-selling things. That being said, this study does push the envelope in presenting a unified approach, whereas previous studies focused on a sub-set of the relevant physics.

Answer:: We agree to the referee’s comment that the previous wording was too broad. We have therefore removed the phrase “for the first time” from the Abstract and replaced it with a more specific statement. What is new in our work is not the general idea of direct-collapse BH formation itself, but the first unified calculation that connects nearby-galaxy irradiation, protostellar heavy-seed formation, and the subsequent obscured rapid growth of the BH within a single framework. Instead of the previous wording, we have slightly modified the last sentence of the abstract to emphasize that our work for the first time demonstrates the emergence of the specific system similar to LRDs consistent with DCBH formation, as “This is the first demonstration that unifies LRDs to a short-lived, enshrouded phase of heavy-seed

formation, which naturally evolving into the overmassive quasars detected by JWST and ultimately the progenitors of today's SMBHs. "

Regarding the methodology, I have two comments/questions:

- It is not entirely clear, after reading the METHODS section, how SN feedback is affecting things. Could the authors please clarify this further? As far as I can see, SN feedback is treated on large scales via a SAM approach (how?), but not locally, in the host systems where the BH seeds form (as all Pop III stars are assumed to have initial masses above 260 M_sun). Again, given the possible disruptive impact of (local) SN feedback, please clarify.

Answer:: We thank the referee for raising this point. SN feedback is treated differently in the source galaxy and in the target halo. In the source galaxy, SN feedback is included semi-analytically in two ways. First, once SNe occur in progenitor haloes, those haloes are assumed to be metal-enriched, and the star formation mode transitions from Pop III to Pop II. Second, for Pop II star formation, SN feedback reduces the amount of cold gas available for subsequent star formation. These are clarified in the line 188 - 191.

In the target halo, we assume that stars with masses in the ranges 8–40 Msun and 120–260 Msun explode as SNe. In our simulation, however, only three stars form in the target systems, namely the progenitors of LBH, MBH1, and MBH2, and all of them directly collapse into BHs without undergoing SN explosions. Therefore, local SN feedback does not operate in these particular target haloes. We have clarified this treatment more explicitly in section Extended Data "Modeling PopIII stars and BHs" at lines 320 - 322.

- The numerical representation of "black holes" are sink particles, with an effective size that is orders of magnitude larger than the actual BH event horizon. That is of course unavoidable, given numerical limitations, but some more direct discussion of this limitation would be good. E.g., the authors show zoom-in results that resolve number densities of 10^8 cc^-3. That is still quite low, in terms of densities where the collapsing gas would reach the opacity limit. How do their zoom-in results compare to figures 5 and 6 in Becerra et al. 2018, MNRAS, 480, 5029 (where higher densities are reached)?

Answer:: We thank the referee for pointing out this important limitation. We agree that our star and BH particles are sink particles with an effective size much larger than the true stellar radii or event horizon, and that our zoom-in simulations do not resolve the final optically thick cores reached in studies such as Becerra et al. (2018). Our aim here is instead to follow the larger-scale and long-term gas supply feeding the protostars and BHs down to the sink scale. Indeed, our finding is similar to the result of Becerra et al. (2018). The accretion rate stays around 1 Msun/yr initially and gradually declines after 10^4 – 10^5 yr after the protostar formation. We have cited the result of Becerra et al. (2018), which shows the increasing numerical resolution does not change the mass growth of the sink particle.
To address this resolution issue, we have added the last paragraph in the section "Zoom-in calculation of seed BH formation in the isolated cloud" (lines 395 - 411).

I understand that Nature imposes limits on the number of references. Still, I would urge consideration of these studies:

- The key effect explored here is the formation of heavy seed BHs in the presence of strong LW radiation. This specific scenario was first proposed in Bromm & Loeb 2003, ApJ, 596, 34 (earlier papers had worked out the importance of LW radiation in suppressing H_2 cooling, but the connection to heavy seed formation was not made).

Answer:: We thank the referee for pointing out this important reference. We had intended to include Bromm & Loeb (2003), but it was inadvertently omitted in the submitted version. We have now restored this reference in the revised manuscript.

- The authors's discussion here of the broad Halpha emission is really nice and convincing. I suggest to cite the recent Rusakov et al. 2026 LRD study in this context (Nature, 649, 574).

Answer:: We thank the referee for this suggestion. We had already cited this work in its arXiv form as Rusakov et al. (2025). We have now updated the citation to the published version, Rusakov et al. (2026, Nature, 649, 574).

- The authors argue that LBH growth is suppressed. That seems quite plausible. But I suggest to briefly compare with two recent related simulation studies (Jeon et al. 2023, MNRAS, 524, 176; and Mehta et al. 2026, Nat Ast, in press). Regarding the former, they argue that LBH growth is suppressed via negative stellar feedback and the "wandering" of light seeds away from the dense gas reservoir; do the authors see similar wandering of their LBH?

Answer:: Yes, this is indeed what we see in our simulation. The gas density around the progenitor Pop III star of the LBH is first reduced by negative stellar feedback. The attached is the figure, which shows the density and temperature evolution around the BH particle, where the gas density stays around 1 cm^-3 and the resulting Bondi accretion rate is below 10^-7 Msun/yr, much smaller than the Eddington accretion rate. Although gas later re-accumulates in the halo, the LBH remains too small to gather and retain a dense gas reservoir, and it wanders in the outskirts of the host halo rather than sinking efficiently to the centre. Because the LBH mass is small, both the Bondi radius and the accretion rate remain low, which also makes dynamical friction inefficient. We have described this in the new section “Inefficient growth of PopIII remnant BH” in the manuscript.

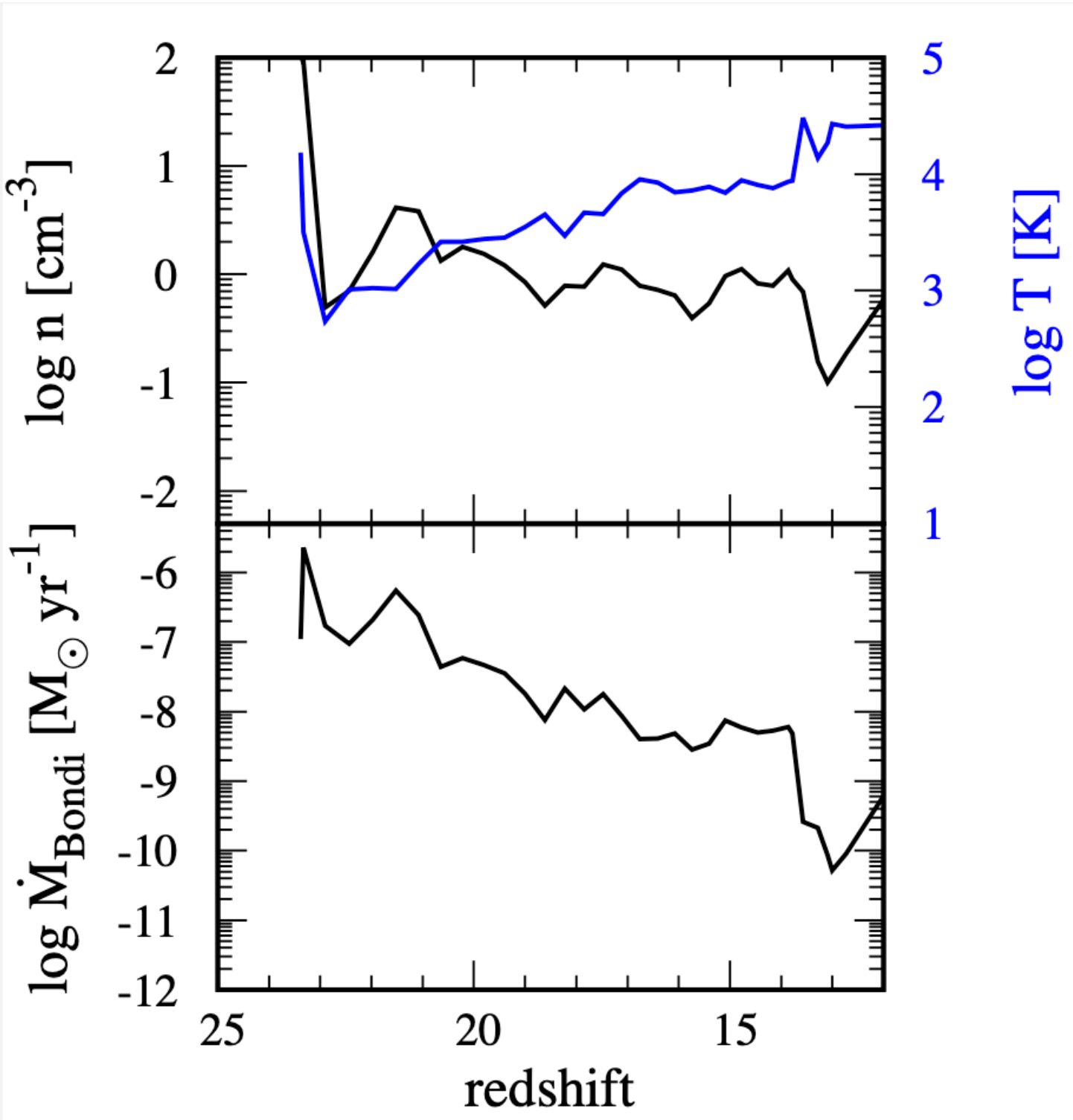

log n [cm^-3]
log T [K]
log Ṁ_Bondi [M_⊙ yr^-1]
redshift
2
1
0
-1
-2
5
4
3
2
1
-6
-7
-8
-9
-10
-11
-12
25
20
15

We thank all three referees for their positive assessment of the revised manuscript and for acknowledging the improvements made in response to the previous reports. Referees 1 and 3 do not raise additional specific issues requiring further changes. Referee 2 raises two remaining points, concerning the definition of the overdensity of the zoom-in region and the omission of non-equilibrium helium chemistry. We have addressed both points in the revised manuscript, as detailed below.

Response to Referee #1

We thank the referee for the positive assessment of our revised manuscript and for supporting publication. We also appreciate the referee's comment that more self-consistent simulations will be required in the future. We agree with this point and have clarified the strengths and limitations of our focused zoom-in approach in the revised manuscript. No further changes are required in response to this report.

Response to Referee #2

We thank the referee for the positive assessment of our revised manuscript and for the two remaining comments.

1. The quoted overdensity refers to the mass overdensity of the selected zoom-in region, not to a galaxy number overdensity. We measured it by computing the mean matter density within the bounding box of the zoom-in region, whose linear size is around 400 comoving kpc, and comparing it with the distribution of mean densities measured for randomly placed regions of the same size in the parent cosmological volume. The selected region corresponds to a 3.8 sigma positive fluctuation in this mass-density distribution. We have clarified this point in the revised manuscript.
2. We agree that helium chemistry can affect the thermodynamics and chemistry of gas exposed to Pop III stellar radiation or radiation from accreting BHs. Helium chemistry becomes important at gas temperatures of several 10^4–10^5 K, where it provides additional cooling channels and increases the free-electron fraction. Although our non-equilibrium chemical network does not explicitly solve helium chemistry, we estimate the ionization state of helium by assuming chemical equilibrium and include the line-cooling rates of He I and He II.

This approximation is particularly relevant for gas irradiated by Pop III stars and accreting BHs. Indeed, Extended Data Fig. 7 shows that radiation from the accreting BH raises the gas temperature well above 10^4 K. The elevated temperature already increases the electron fraction and thereby promotes H_2 formation; including full non-equilibrium helium chemistry would likely further enhance the H_2 abundance. However, we expect our main conclusion to remain unchanged. Even if enhanced H_2 cooling lowers the cloud temperature, the high inflow rate is sustained and still allows the formation of MBH2. We have added this discussion to the revised manuscript.

Response to Referee #3

We thank the referee for the positive assessment of our revised manuscript. We are pleased that the referee finds the methodology, assumptions, caveats, and novelty of the study to be clearly presented in the revised version. No further specific changes are requested in this report.
